# Inflammations of the Liver and Their Sequelœ, &c.

**Published:** 1886-06

**Authors:** 


*Inflammations of the Liver and their Sequelæ, &c.* By Dr. GEORGE HARLEY, F.R.S. London: J. & A. CHURCHILL. 1886.

Although a small medical work by an author of repute can seldom have been prefaced by so much bad grammar and worse taste, a conscientious perusal of the book itself shows that Dr. G. Harley's manner does some injustice to his matter, and that his extensive experience in diseases connected with the liver has enabled him to make some suggestions and tabulate some facts, which, if not absolutely new, are at least freshly put forth, and go far to prove the propositions he maintains. This is well shown in the statistics he quotes to show that hepatic abscess is much less infrequent in England than has generally been supposed, and, indeed, is more common in Englishmen in their own country than in those residing in India! As Dr. Harley lays claim to "practical and theoretical innovations," it is curious that when he says so much upon the pathology of the varieties of hepatic abscess, no allusion is made to the novel fact, that many of the cases of diffuse suppuration of the liver occurring in this country are found to be examples of Actinomycosis, if they are subjected to microscopical investigation.

A paragraph in the earlier part of the work raises hopes that some really new methods of treatment will receive full description and discussion: these are, direct hepatic phlebotomy, puncturing the liver capsule in congestive hypertrophy, and emptying the gall-bladder of calculi by means of manual and digital kneading. Of each of the operations of puncturing the capsule and of direct hepatic phlebotomy, one example only is given; but of the latter necessarily hazardous operation physicians would like to have more of Dr. Harley's experience recorded before they will feel themselves justified in putting it into practice. Of the method of removing gallstones from gall-bladders, and calculi from ureters in which they are impacted, by the process of kneading, no examples or details are given, though the treatment has been practised by Dr. Harley "successfully during the last few years." Until conclusive evidence can be adduced to prove that it is possible to move a calculus along the duct by kneading the abdominal walls, most persons will consider that the procedure is at the best but useless.

With Dr. G. Harley's large opportunities we should expect a more philosophical treatise than is this book, in which Self seems more prominent than either Syntax or Science.

